# Summary of Sloan symposium: healthy buildings 2015-Europe

**DOI:** 10.1186/s40168-015-0115-4

**Published:** 2015-12-02

**Authors:** Hal Levin, Martin Täubel, Mark Hernandez

**Affiliations:** Building Ecology Research Group, Santa Cruz, CA, USA; Living Environment and Health Unit, National Institute for Health and Welfare, Kuopio, Finland; Department of Civil, Environmental, and Architectural Engineering, University of Colorado, Boulder, CO, USA

**Keywords:** Microbial ecology, Indoor environment, Practice, Research agenda, Symposium

## Abstract

The Sloan Symposium, “Microbiology of the Indoor Environment,” was held to facilitate dialog on biological research between scientists and practitioners in the field which was complementary to the adjoining activities of the ISIAQ Healthy Buildings Europe conference, Eindhoven, Netherlands, on 20–21 May 2015. Multi-media archives of these special adjunct proceedings are presented.

**Electronic supplementary material:**

The online version of this article (doi:10.1186/s40168-015-0115-4) contains supplementary material, which is available to authorized users.

## Summary

Excess or abnormal microbiological activity is both a cause and a consequence of unhealthful indoor environments. Building research, investigations, and remediation must identify compromised buildings in a prioritized manner to avoid potential harm to occupants. Currently, there are no good quantitative and objective criteria for determining and ranking either the status of buildings or the efficacy of remediation from a microbial perspective. In extreme cases of severely damaged buildings this lack of objective and quantifiable criteria is not a practical problem. However, there exists a large middle ground where consultants and remediation professionals would welcome criteria to rank and prioritize building conditions and to state the efficacy of methods used to address identified or potential problems. Classical microbiology is currently and effectively applied in qualitatively assessing the microbial condition of buildings. An important question is whether and which- molecular methods might be incorporated to aid in the diagnosis of a building’s “microbial status”. The use of high throughput DNA sequencing methods raises important questions as to how to interpret the results of ecological approaches to indoor environment research or applications.

## Introduction

The Sloan Symposium on the Microbiology of the Indoor Environment (MoBE) was held in Eindhoven, Netherlands, in cooperation with the Healthy Buildings 2015-Europe^1^ Conference on May 21–22. The symposium was designed to introduce and cross-fertilize emerging applied biology with the building science community, and to improve understanding of the potential uses of molecular biology methods by building scientists and practitioners. During the symposium, we attempted to identify key research questions based on practical needs that can lead to more widespread and contextually appropriate use of molecular methods and collection of useful metadata.

This special symposium consisted of a plenary lecture, two technical sessions (eight papers), and an interactive workshop facilitated as an open forum embedded in the Healthy Buildings 2015-Europe conference program followed by an Annex Workshop to capture, expand, and to prioritize potentially important future indoor environment research themes (details are provided in Additional file 1). Both activities were supported by the Alfred P. Sloan Foundation as part of the Microbiology of the Built Environment (MoBE) Program^2^ through a grant to International Society for Indoor Air Quality and Climate (ISIAQ)^3^.

## Background

The symposium presented and addressed barriers to the widespread practical use of the latest molecular microbial ecology methods to characterize indoor environments, in an effort to ultimately help practitioners to use these data in creating more healthful settings. Some professionals in attendance suggested various potential and common microbial contamination situations including but not limited to (1) mold growth associated with water damage; (2) targeted pathogen surveys; and, (3) wide area surveillance of indoor microbiomes driven by adverse occupant health reports.

There was general consensus that while the applied microbiology developments emerging in this research community—first and foremost, DNA recovery methodology and in particular, next generation sequencing—have had notable impacts as judged by common academic metrics; however, these advances have not successfully translated into paths which are available for practitioners to apply such methods or interpret these results with confidence in the field.

Practitioners voiced a need for the cohorts of emerging research methods to be vetted with quality assurance of their reproducibility in practical contexts, and raised concerns regarding associated liabilities and litigation potential. The absence of simple and practical paths for interpretability of next-generation sequencing data was duly noted, and it was suggested that the quality of reference data currently make these methods non-applicable in building investigations outside pure research arenas. A salient theme emerged as to how relevant agencies, public health officials, as well as building owners, operators, and occupants should understand the short- and long-term risks and implications of applying emerging molecular methods in practical context prior to future implementations.

Until relatively recently, microbiological investigations of indoor environments depended largely on the record of recovery of culturable microbes and conventional light microscopy where results have inherent variabilities from sampling and operator subjectivities. While representative sampling remains an issue, the introduction of molecular microbiology methods has increased the resolution for identifying microorganisms in the environment by an average factor between ten and a hundred during the past decade, while the associated analytical costs have dropped to the level of conventional microbiological analyses. The huge cost reduction in genetic analyses, and expansion of their use, has been accompanied by increasing attention from practicing sectors—applied research and consultants. Accompanying this growing attention has been a substantial caution for better understanding the strengths and limitations of these methods, the field preparation, and laboratory processing resources, as well as confidence in practical use of results, with a focus on actual interpretation of data sets.

While microbial ecologists report major findings as relative abundance, associations, and attribute sources by leveraging probabilistic models, practitioners have to relate these findings to an open-ended network of other indicators in many different environmental contexts, and constantly confront associated errors and omission risks.

Today, much microbial work supporting building investigations is still done using traditional, culture-based methods and microscopy, which clearly indicates that the threshold for using advanced molecular methods outside the science applications is high in this field. This threshold may in part be defined by both, limitations of the novel molecular methods (e.g., the lack of reference data) and strengths of the traditional methods (e.g., the fact that cultivation, with all its limitations, does provide surrogate indications of what and how much could be “alive” in a building). Next-generation sequencing still largely fails to identify viability. This situation anticipates change: once confident translation to practical implementation and interpretation has been made for molecular applications—building characterization and engineering interventions—there can be sustainable use of molecular methods to study the indoor environment. The resilience of environmental DNA data in the litigation environment, however, has yet to be determined. Issues such as recovery and the difficulties in actually interpreting results from new molecular applications, with the lack of reference data on the one hand, and actual health-interpretation of microbial community analyses on the other, became very evident.

## Results and discussion

### Summary of discussion points

- Practitioners need confidence in their ability to provide clientele with results that non-experts can understand.
- MoBE requires more integration with other branches of building science:
  - □ Chemistry of air and surfaces and water infrastructure
  - □ Microbiology of air and surfaces and water infrastructure
  - □ Tools to (affordably) analyze building characteristics and occupant interactions
  - □ Dynamic models and analysis

- Given generalized states of building design, material stability and microbial response to water activity, resources might be better spent on building moisture dynamics and their reliable and standardized measurement than on further characterization of the indoor microbiome:
  - □ Increased networking between indoor air scientists, practitioners and microbial ecologists could improve the quality and utility of study results.
  - □ Increased understanding of the obstacles to wider use of molecular biology methods.
  - □ Recognition of the need for condensing methods to practical, reproducible terms, which can confidently lead to summary information on the environmental health risks presented by the condition and operation of a given building.

- Recognition that indoor microbiome studies conducted thus far have by-and-large not been done to aid translation to practice for sampling, analysis, and interpretation of results. To address this will require more research with blinded trials, leading to compilation of guidelines and standards for robust field applications of molecular biology methods.
- Some research has confirmed that people are dominant sources of bacteria and fungi indoors and that people’s behavior has an impact on indoor microbes; thus, not only building factors, but also building use, maintenance and its occupants need to be considered when measuring and intervening with indoor microbiota

### Synthesis of some of the major themes

- Buildings are systems and interstitial spaces (inside walls, ceiling, and floor cavities) are important.
- The role of moisture in adverse building outcomes is clear—do/should we care about anything else?
- Microbial methods: are there broad approaches (e.g., ATP) that offer as much value as molecular methods?
- Molecular methods need to converge towards quantitative interpretation (including locally applicable reference data) and dead/alive differentiation.

### Selected research topics that may deserve future attention

- How do buildings increase or decrease microbial diversity, what is the hierarchy of building factors with impact, and how relevant is diversity to start with?
- Microbial hot spots in buildings: map where the microbial metabolism actually occurs in a building.
- What bacteria grow in buildings and to what extent does this growth correlate/co-occur with fungi?
- What is the health relevance of the indoor microbiome—do we know enough to evaluate this question, or do we rather make wild guesses?

## Endnotes

^1^Alfred P. Sloan Foundation Microbiology of the Built Environment Program (http://www.sloan.org/major-program-areas/basic-research/mobe/)

^2^International Society of Indoor Air Quality and Climate (ISIAQ: http://www.isiaq.org)

^3^Healthy Buildings 2015-Europe conference (http://hb2015-europe.org/)

## Additional file

Additional file 1:
**Supplementary information.** (DOC 86 kb)

