# Supplementary material for: Summary of Sloan symposium: healthy buildings 2015-Europe
Source: Microbiome. 2015 Dec 2;3:68. doi: 10.1186/s40168-015-0115-4 (PMC4667518; doi:10.1186/s40168-015-0115-4)
Supplement: Additional file 1: — Supplementary information. (DOC 86 kb) [file 40168_2015_115_MOESM1_ESM.doc]

# SUMMARY OF SLOAN SYMPOSIUM: HEALTHYBUILDINGS 2015-EUROPE

# Supplementary Information

**Hal LEVIN^1,4^, Martin TÄUBEL^2^, and Mark HERNANDEZ^3^**

Hal Levin, Building Ecology Research Group, Santa Cruz, California.HLevin6@gmail.com

Martin Täubel, National Institute for Health and Welfare , Kuopio, Finland. Martin. Täubel@THL.fi

Mark Hernandez, Department of Civil, Environmental and Architectural Engineering, University of Colorado, Boulder, Colorado. Mark.Hernandez@Colorado.edu

**^*^Corresponding email:** [**HLevin6@gmail.com**](mailto:HLevin6@gmail.com)

**Supplementary Information**

**Overview of the symposium**

The Symposium began with a plenary lecture by Dr. Miia Pitkäranta, relating a composite of Finnish research and practitioners perspectives (summarized in SI). This was followed by eight presentations, and a short discussion at two successive technical sessions and workshop formally embedded within the conference. The oral presentations included overviews of sampling, molecular analyses, and applied microbial ecology as primers for non-experts.

See Table 1 for topics, presenters, and links to on-line video archives.

**Table 1. Presenters of papers and paper titles All videos can be viewed at** [**http://isiaq.org/hb2015_europe_sloan_symposium.php**](http://isiaq.org/hb2015_europe_sloan_symposium.php)

| ***Presenter*** | ***Presentation title*** |
| --- | --- |
| Miia Pitkäranta | Molecular Tools and Microbial Ecology of Buildings - A Practitioner’s View |
| Jordan Peccia | Revolution/evolution—DNA sequencing to identify indoor microorganisms |
| Jeffrey Siegel | Building Science and Indoor Air Determinants of the Indoor Microbiome |
| Anne Hyvärinen | Assessment of moisture and mold problems – the Finnish example |
| Maria Nunez | Microbial sampling in building surveys: what and why are we sampling? |
| Tiina Reponen | Microbial sampling in building surveys: how to choose a sampling method? |
| Martin Täubel | Quantitative PCR in microbial assessments of indoor spaces. |
| Mark Hernandez | A Perspective on Leveraging New Generation Sequencing for Bioaerosol Assessments of the Built Environment: Differences and Commonalities of Processing Pipelines and Databases |

**The Conference Workshop** followed immediately after the technical sessions. The purpose of the Conference Workshop was to gather input from practitioners on the barriers and challenges they face in adopting molecular methods for characterizing indoor microbiomes. The workshop video can be viewed at <http://isiaq.org/hb2015_europe_sloan_symposium.php>

The Annex Workshop also included a focus on scientific issues which were identified as “translational barriers” during the Conference Workshop, as well as issues raised by the assembled experts. The participants included the plenary lecturer, the 9 invited presenters (plenary and technical sessions) and 5 additional experts considered as though leaders in the field including Jack Gilbert, Ulla Haverinen-Shaughnessy, Paula Olsiewski, David Thaler, Inge Wouters, and Hal Levin, facilitator. The Annex Workshop began with a discussion of biodiversity based on presentations by Jack Gilbert and Jordan Peccia.

Dr Gilbert presented work on the analysis of microbial communities in numerous built environments, including homes, hospitals, restrooms, and gyms. The focus of his presentation was on the key influence of these microbial ecosystems on human health.

"Dr. Peccia presented previous work from his lab and others that identified specific taxa and microbial community parameters which are associated with human health. This included the relationship between microbial richness and asthma/atopy development, and listing bacteria and fungi that have been associated with asthma severity. A framework was then presented to apply next generation DNA sequencing data to both identify these health-based targets and for unraveling how building variables modulate human exposure.

1. **Technical presentations**
   1. **Conference Plenary lecture**

# MOLECULAR TOOLS AND MICROBIAL ECOLOGY OF BUILDINGS - A PRACTITIONER’S VIEW

Miia J. Pitkäranta, Building physics expert services, Vahanen Group, Espoo, Finland

The microbiological research of the built environment (BE) aims at understanding the microbial processes in building structures and indoor environment and their potential impact on the users’ health. This knowledge is needed in new building design and building physical modelling, choosing effective remedial actions in old, microbially damaged buildings and controlling the success of remediation. Also, the development of diagnostic protocols that serve practical building investigation purposes rely on this knowledge.

Molecular tools, i.e. laboratory methods that assess microbial DNA, RNA or proteins are increasingly used in microbial ecology studies, including studies of the indoor environment. Certain molecular protocols have also been adopted and validated for use in practical building assessment. The presentation will discuss the present “molecular toolbox”, its major capabilities and pitfalls, as well as some useful molecular methods not commonly used in BE studies. The key findings and conclusions inferred from the recent molecular indoor microbiome studies are described briefly.

The concept of “microbial ecology of a building” is discussed from the perspective of the traditional building microbiology and IAQ problem buildings. It is stressed that in the majority of buildings significant microbiological activity does not occur in the indoor air, dust or surfaces. Outside obvious wet locations such as drains, sinks and dish washers, the most common sites where sufficient moisture may exists to actually support microbial colonization, proliferation and community development include internal structural parts susceptible to leaks, condensation, rising damp and slow drying. The question is raised whether knowledge about the common building structures and materials, prevailing physical conditions and associated moisture risks is necessary for a microbiologist whose work addresses buildings as microbial habitats.

Various aspects of microbial growth on building materials have been assessed in the past using viable methods. However, several issues that are of practical interest but have not been well characterized will be highlighted. Many of these require or would greatly benefit from the use of molecular tools. These include e.g. the characterization of the diversity of bacterial communities growing on various building materials, of long term dynamics of mixed microbial communities and of natural microbial populations thriving on aged, originally mold and microbial resistant materials. It is suggested that research on air and surface should in the future be complemented by research of microbiomes on building structures and materials.

While microbes play an important role in indoor environmental health issues, it is notable that in problem buildings microbiological contamination is commonly accompanied by other factors such as chemical emissions and physical impurities, which may partly originate from the same moisture damaged sites as microbial contaminants. The research needs for elucidating the role of microbial degradation in material emissions, and the health effects of combined exposures are discussed briefly.

- 1. **Technical session presentations**

**ABSTRACTS FOR TECHNICAL SESSIONS (link to videos of all presentations available at** [**http://isiaq.org/hb2015_europe_sloan_symposium.php**](http://isiaq.org/hb2015_europe_sloan_symposium.php)**)**

***Jordan Peccia*** (USA^1^Dept. of Chemical and Environmental Engineering, Yale University, New Haven, USA

**Revolution/evolution—DNA sequencing to identify indoor microorganisms**

Over the last 10 years, biology has been revolutionized by the ability to sequence DNA rapidly and inexpensively. Using prior taxonomic and phylogenetic frameworks, the indoor microbiology field has thus far leveraged next generation DNA sequencing to build catalogues of indoor microorganisms, defined relationships between building variables and microbial communities, and has made limited strides in understanding how indoor bacteria and fungi impact human health.

This talk provides a DNA sequencing methods overview for the Healthy Buildings Conference Annex Workshop on Microbial Characterization. It will provide perspective on the *revolution* of next generation DNA sequencing, and its *evolution* and potential for indoor microbiology research and applications.

An overview of next generation DNA sequencing, phylogenetics, and computational tools will first be presented. The talk then describes next generation sequencing benefits, including the expansion of microbial taxa that can be considered in building studies, and current limitations, including limited quantitativeness, uncertainty in taxa identification, and issues with identifying rare, but important taxa. Case studies will be presented on the computational approaches for incorporating next generation sequencing data into human health studies. Finally, a future vision is presented for extending DNA sequencing of indoor samples into metagenomics analysis for identifying bacterial and viral pathogens and conducting microbial disease epidemiology.

Incorporating next generation DNA sequencing methods into building microbiology and health studies has the potential to greatly increase our view of the quantity and diversity of microbes in buildings. Current taxonomic and phylogenetic approaches should be continued and quantitativeness improved. Adding metagenomic methods to identify viral and bacterial pathogens can improve our understanding of microbial disease transmission in buildings.

The microbiology of the built environment is complex. The application of next-generation DNA sequencing technology is able to capture this complexity and holds significant promise for unravelling relationships between building design and operation, human health, and microbial communities.

***Jeffrey Siegel*** (USA) Department of Civil Engineering and Dalla Lana School of Public Health, University of Toronto

**Building Science and Indoor Air Determinants of the Indoor Microbiome**

The past decade has revealed rich and diverse indoor microbial communities. Although we see profound differences within and between buildings, the underlying mechanisms that lead to these differences and the role of indoor environmental factors remain relatively unexplored. Some specific research questions include.

1. What building science parameters need to be addressed? Is the commonly measured air relative humidity sufficient, or do we need to measure surface or material moisture? Are the presence of occupants the main descriptor of occupancy or is occupant activity more relevant? Is knowledge of the surface material sufficient or is the composition and amount of soiling needed?
2. How do building science parameters interact with each other from the perspective of influencing the microbial community? Are there likely to be combined effects (for example the impact of moisture and temperature together) that should drive measurement approaches?
3. With what frequency and for what duration do we need to measure building science parameters? Are short-term measurements sufficiently predictive of microbial community change for some parameters? Which building and indoor air parameters are sufficiently static that a single or infrequent measurements are sufficient for characterization?
4. For what building science parameters are average values appropriate descriptors and for which parameters are extremes or duration/amount of time above a threshold more relevant?

The overall purpose of this presentation is to use the existing literature as well as several large publically-available building science datasets to answer these questions as well as explore needs for future research.

***Anne Hyvärinen*** (Finland)

**Assessment of moisture and mold problems – the Finnish example**

|  |  |
| --- | --- |
| The Finnish Health Protection Act considers moisture and mold damage in a building as circumstances that may affect negatively on health and are thus treated as a potential health hazard that need to be removed. The responsibilities for occupants, building owners and health authorities within the process of recognition, reporting, investigation and remediation of moisture damage are defined. Guidance on how to assess moisture and mold problems, and the role of microbial measurements, are discussed.  An assessment of a building for moisture and mold damage should consider collection of information on technical aspects and the history of the building, a technical inspection including evaluation of moisture and mold damage, microbial and other measurements (also such done earlier), symptom questionnaires, the probability of exposure, and summary of results, interpretation and reporting to building occupants, owners and where needed authorities.  The Finnish Healthy Housing Guidelines provide information on methods and procedures, and on the interpretation of results for various relevant indoor parameters, including microbial growth and contamination. While not being heath-based, guidance is given what is normal, and what might indicate an abnormal microbial situation. Sampling for microbes and analyses of samples are part of an entity, sources/conditions for potential health hazard and probability of exposure are determined. A ‘holistic’ building assessment requires knowledge on the history and technical details of buildings, and implies a detailed technical building investigation, where e.g., moisture damage and their causes, risk structures and ventilation are assessed and the probability of exposure is concluded.  Recommendations for actions in situations of moisture and mold damage in buildings should be based on the entity of a comprehensive assessment. Microbial measurements may be used to contribute to such assessment, but should not be used as a single or the sole assessment tool. | |

**Kristian Fog Nielsen (Denmark)**

**Microbial growth and interactions on indoor surfaces - microbial secondary metabolites and mycotoxins**

|  |  |
| --- | --- |
| Fungal growth indoors is a big problem and with weather changes and the current push towards higher energy efficiency, material recycling, and reduction in constructional costs, the frequency of microbial problems indoors will increase. Fungi require organic material for growth and are capable of growing on almost any material, also totally inorganic materials after these have been contaminated by organic matter e.g. during construction or via flooding. Most fungal species produce numerous secondary metabolites needed in their natural habitats. Some compounds are aimed at bacteria, other fungi or predating mites and insects. Some of these metabolites, the mycotoxins, may interfere with the human receptors body and result in undesired toxic effects.  Fungi needs to adjust the internal water activity (a_w_, ≈local RH) to the surrounding a_w_ by producing internal solutes like polyoles (glycerol, trehalose). This requires lots of energy, resulting in decreased growth rate and often lower production of secondary metabolites.  *Chaetomium, Aspergillus, Stachybotrys*, which are associated with the worst adverse health effects, produce high amounts of mycotoxins and have therefore been suggested as one of the major causes of the adverse health problems. However, conclusive evidence, including exposure-response-time relationships and differences between individuals, are not available.  Most toxic fungi require almost liquid water for growth and mycotoxin production, however, this is also conditions where bacteria thrives and can produce toxic and immuno-modulating compounds. Few secondary metabolites have been evaluated for their inhalative toxicity which in most cases will be much more potent than oral toxicity (many compounds may only be toxic through inhalation). Exposure to mycotoxins is thought to be particle-borne as they are not considered volatile, and thus most exposure may occur via inhalation of spores and especially micro-particles (<1 µm) liberated from desiccated, decaying fungal matter. | |

***Maria Nunez* (Spain, Norway)**

**Microbial sampling in building surveys: what and why are we sampling?**

Buildings are the main arena where human life unfolds. Numerous microbial spores and fragments from both the outdoor environment and building occupants (humans, their pets and plants) settle on surfaces, while others become airborne or are removed by surface cleaning and steadily intricate HVAC systems. There is always a background level of microbial fragments and spores in buildings. But do buildings have their own microbiome? And what happens when this microbiome is altered?

Sampling in sick buildings can serve to several purposes. For mould remediation, sampling may be unnecessary if visible mould damage is present. In cases of hidden mould, sampling should focus on finding microbial sources within the building, i.e. species that are capable of growing and proliferating after moisture damage, and not microbial sinks (settled fragments from other sources, not proliferating). A simple tape lift can document *in situ* mould growth on building structures and materials. Indirect methods as air sampling can be useful to detect hidden mould, if both building physics, and the biology and ecology of the species involved are well known. For epidemiological studies, DNA methods are capable of identifying exposure agents out of small, sterile microbial fragments.

Different sampling and identification techniques can address different challenges related to the indoor environment. We provide practical examples of sampling for different purposes.

In order to address building issues, we need to characterize the building microbiome in every case, and critically contrast it with other microbiomes sharing the same building environment. Differentiating microbial sources and sinks is necessary in order to set the building microbiome back to normal levels. Cleaning and disinfection of surfaces is not an effective measure when the surface is a microbial sink, and not a source.

The choice of adequate sampling strategies indoors requires differentiating microbes capable of growing in buildings (the building microbiome) from those accidentally encountered in buildings, unable to replicate. A thorough consideration about the purpose of indoor sampling will minimize building intervention, occupant burden, and costs.

**Tiina Reponen** (Finland, USA) , Department of Environmental Health, University of Cincinnati

**Microbial sampling in building surveys: how to choose a sampling method?**

The choice of a sampling method depends on the purpose of the sampling: is sampling conducted for verification of the presence of microbial problems, identification of the source, monitoring the efficiency of control methods or assessment of the human exposure. When choosing a sampling method, one also has to consider which analysis method would give most relevant measures for the question asked. This in turn may limit the choice of sampling methods available.

A wide variety of sampling methods are available for assessing microorganisms in indoor environments. These can be categorized in four main groups: air sampling, dust sampling, surface sampling and building material sampling. This presentation will review the traditional and modern sampling techniques available and will discuss the advantages and disadvantages of the choices available.

Air samplers are based on well-established aerosol collection mechanisms, such as impaction, interception, diffusion, electrostatic attraction, and gravitational settling. The same physical principles that are applied to non-biological particles can be applied to bioaerosol sampling in terms of sampling efficiency of a given particle size range. Additional consideration for sampling microorganisms is the ability to maintain the biological property that is used in the analysis, e.g., culturability of cells or integrity of genetic material. There are also a few direct-reading instruments, most of which are based on laser-induced autofluorescence of biological material. Dust sampling can be done by vacuuming the surfaces using various collection devices attached to the vacuum or the sampling pump. For both air and dust sampling, some practical considerations include the noise level, need for electrical power and the weight of the equipment. Surface sampling methods include swab sampling, tape lifts and contact plate sampling, whereas building material sampling simply means collecting pieces of materials into clean containers.

As each technique has unique advantages and disadvantages, it is often beneficial to use multiple techniques in each investigation. This way, the limitations of one technique can be are compensated by another one.

Sampling for microorganisms should be designed so that it complements visual assessment in a meaningful way. Practitioners need to understand the underlying limitations of the methods they are using for microbial sampling in order to properly interpret the results.

**Martin Täubel** (Austria, Finland) Finnish National Institute of Health and Welfare , Kuopio, Finland

**Quantitative PCR in microbial assessments of indoor spaces.**

Microbial contamination of building materials, surfaces, or indoor air is traditionally assessed using cultivation technique in support of building investigations. In research settings, the amount of specific cell wall components of bacteria and fungi are being measured in addition to assess indoor exposure. Both in practical situations as well as in research settings, a shift towards DNA-based approaches is taking place. Here, we discuss quantitative PCR (qPCR) as a method to assess indoor microbial contamination.

QPCR analyses were performed in parallel to cultivation for fungi and bacteria from building materials. Active air samples were collected from homes with and without moisture damages and from outdoor locations, and microbial determinations were performed both with qPCR and cultivation. The effects of using different standard curves and internal standard on qPCR results are simulated.

Results from cultivation and qPCR carried out in parallel from building material samples correlate moderately (e.g. Spearman correlation coefficients for total fungi and *Penicillium*/*Aspergillus* group between 0.61-0.68). QPCR values are typically many times higher compared to cultivation results, due to detection of both non-culturable and dead fungal cells/fragments in qPCR. Depending on cut-offs defined for qPCR results, a part of samples that are categorized as ‘non-contaminated’ based on cultivation will be categorized as ‘contaminated’ using qPCR. QPCR targeting total fungi and *Penicillium/Aspergillus* group from air samples seems to discriminate homes with from homes without moisture damage better compared to cultivation.

Due to quantifying different spectra of the microbial content within a sample, there is no perfect agreement between cultivation and qPCR methods. As with cultivation, it will be crucial to provide detailed guidance on sample processing and the conduction of the actual qPCR measurement, if guidance values should be provided for the use of qPCR in indoor microbial assessments.

In an era where microbial determinations shift from cultivation to DNA-based approaches, knowledge is needed on the weaknesses, strengths, and especially the peculiarities of these new methods, in order to facilitate their application beyond research. This work discusses such aspects for the quantitative PCR method.

**Mark Hernandez** (USA) Department of Civil, Environmental and Architectural Engineering, *University of Colorado, Boulder, Colorado, USA, 80309*

**A Perspective on Leveraging New Generation Sequencing for Bioaerosol Assessments of the Built Environment: Differences and Commonalities of Processing Pipelines and Databases**

A number of recent built environments studies have used “next-generation” high throughput sequencing to characterize surface associated and airborne microbial communities in residential and commercial buildings. Along with ultra-clean sampling methods need for this forensic approach comes the requirement for substantial computational power, tailored software, relational databases, as well as modest computer skills. Once samples are acquired, they can now be outsourced to specialty laboratories for sequencing and reports on the relative abundance of the microbes collected―as would conventional aerobiological samples.

Most of these high throughput analyses target and report relative abundances using the most accepted evolutionary genes used for “identifying” bacteria and fungi; however, the data analyses from these types of studies can be tenuous because of a complicated conglomerate of “ubiquitous (processing) artifacts”.

Given the rapid maturation and availability of “high throughput sequencing” we present here the range of processing pipelines and databases which are now available to the science, engineering and industrial hygiene community for this purpose. We present the basic computational tenants of the major pipelines, as well as the characteristics of the relational databases they use for determining the relative abundance in surface and aerosol samples recovered from the built environment.

Ultraviolet laser induced fluorescence (UV-LIF) is gaining increased attention for its ability to characterize intact airborne microbes. However, there is a lack of calibrated spectral libraries archiving the optical signatures of bioaerosols commonly present indoors. A method for the interrogation of aerosolized pure microbial cultures was developed for the express purpose of obtaining characteristic optical responses of model bioaerosols from Wideband Integrated Sensors (WIBS). A research grade WIBS instrument was challenged in environmentally controlled chambers (1m^3^ and 10m^3^) with pure cultures of bacteria and fungal spores and – at different stages of growth. From this challenge, fluorescent spectral libraries were constructed for 36 fungi that comprise the analytical targets in the US Environmental Protection Agency ERMI (Environmental Relative Mold Index), as well as 6 common pathogenic bacteria models, all of which collectively serve as important model bioaerosols that are germane to indoor aerosols and public health.

As judged by optical diameter, light scattering and relative intensity emission patterns, common airborne fungi fall into 4 major, and two minor, optical groups, where the ratio of 280/370 excitation response in the 420-650nm excitation is the most sensitive optical arena. Bacteria divided into two distinct groups.

Databases of disaggregated airborne particle observations by room and structure, from more than 2000 composite scans of the interiors of water damaged structures, were compiled from 6 major metropolitan areas. Bioaerosol particle assignments in physiologic groups as determined by chamber studies and normalized to concurrent outdoor samples were rank ordered to form log-normal distributions of total particle concentrations and mold-like particle concentrations. Real-time observations were then juxtaposed to their contextual percentile rank suggesting their conformance, or position as an outlier.

Optical spectra associated with bioaerosols can thus be compiled into useful databases and distributions. These may be leveraged―in real time―to denote significant deviations from indoor air norms. These results suggest that multi-channel LIF may be leveraged into a reliable forensic platform to monitor bioaerosols.

1. **Conference Workshop**

See video of workshop at [**http://isiaq.org/hb2015_europe_sloan_symposium.php**](http://isiaq.org/hb2015_europe_sloan_symposium.php)

1. **Annex Workshop summary**

**Summary of the A.P. Sloan funded Annex workshop at Healthy Buildings Europe, Eindhoven, 20.-21.05. 2015**

Participants:, Jack Gilbert, Alina Handorean, Ulla Haverinen-Shaughnessy, Mark Hernandez , Anne Hyvärinen, Hal Levin (facilitator), Kristian Fog-Nielsen, Maria Nunez, Paula Olsiewski, Jordan Peccia, Miia Pitkäranta, Tiina Reponen, Jeffrey Siegel, Martin Täubel, David Thaler, Inge Wouters

The objective of the Symposium Annex Workshop that had been defined prior to the Healthy Buildings conference in Eindhoven was to identify the key scientific challenges to assist practitioners in the use of culture independent methods and in the interpretation of results from the use of these methods. The activities that had been supported by the A.P. Sloan foundation in the course of the HB 2015 were streamlined such that in the conference workshop on Wednesday afternoon we were seeking to get key input from practitioners on the issues they are facing when trying to incorporate the molecular methods into their work. That did not happen to an extent that we were hoping for, which is why we had to adapt the activities in the Annex Workshop accordingly.

Within the group of participants of the Annex Workshop, consisting of scientist from different fields all linking to MoBE activities, including microbiology, engineering, epidemiology, biochemistry, as well as building practitioners, the Annex Workshop started with a brain-storming of what the group may identify as research topics that would deserve attention in future activities of the MoBE program. The list of proposed topics / study questions in the following is reduced to those topics that were considered most relevant by the Workshop participants and that got selected for further, more detailed discussion, which is briefly summarized in bullet points under each topical header.

- **How do buildings increase or decrease microbial diversity, what is the hierarchy of building factors with impact, and how relevant is diversity to start with?**
- We are now modifying the indoor microbiome in a more or less random way. If we are able to identify those processes that determine diversity of the indoor microbiome, we can provide a tool kit to better operate and design buildings.
- Can we apply probiotics inside buildings to improve the indoor microbiome? We would have to balance existing building frameworks and modified, immune-stimulating indoor environments.
- Buildings are notoriously complex (and the built environment does not only include homes!) – how do we identify the key processes in this context?
- Example: farming and elevated microbial diversity indoors: is the building microbiome as such really relevant, or is it more the environmental circumstance in which the building is located?
- What are the experiments we’d want to design to answer those questions?
- Only change diversity of the indoor microbiome and control all other factors … use test home.
- Bring animals into these test buildings. Study their microbiome and figure out what is healthy.
- Do interventions in real buildings that increase diversity, as much independently from other indoor environmental variables. Key questions are: how do we increase diversity and are we even able to control such intervention? And what are the health endpoints one should be looking at?
- Study the microbiome and its diversity on different building materials under ’normal’ conditions – map and create a database.
- **Microbial hot spots in buildings: map where the microbial metabolism actually occurs in a building.**
- It would be crucial to also sample inside structures and characterize the microbiome in those spaces, along with chemicals and other, physical parameters. Monitor microbial dynamics in structures over time. Semi-micro-environmental studies. Take into account pressure difference, air flows.
- Following approaches could be followed:
  - Map moisture: predict moisture based on RH and T differences – modeling is existing, could be done at the stage of blueprints. But there are obvious limitations ..
  - Metabolic activity in the sense of reductive activity (dye): Scan a building surface for fluorescence illumination to map growth/colonization
  - Chemical approach (GC …), volatiles
  - Microscopy, spore production
- **What bacteria grow in buildings and to what extent does this growth correlate/co-occur with fungi?**
- Fungi/Bacteria co-association studies, carried out in different countries/climates/buildings: top 10 fungi - what bacterial communities are abundant in a sample that is dominated by fungus x?
- Quantitative sampling should be included that takes into account the surface area. The type of materials matter!
- Representative sample from a surface? What is a surface?
- Analyses layers in drilled core sample for (live) bacteria
- **What is the health relevance of the indoor microbiome – do we know enough to evaluate this question, or do we rather make wild guesses?**
- Differentiate what is:
  - *current practice:* avoid/repair leaky buildings; remediation, abatement.
- *best practice based on current knowledge:* chose materials well?
- *what are immediately achievable goals with slight modification (based on scientific knowledge):* Bacteria mate Fungi: understand better bacteria that are associated with the relevant building fungi, explore health impact
- what are futuristic, imaginative scenarios (that may lead us towards the microbial neolithic revolution):
  - Reconstruct the way people build houses
  - Low microbial growth buildings
  - Probiotics in buildings; Fecal microbiome transplants for buildings (make sick buildings healthy); Inoculated building materials: spores that won’t grow until they get wet, but when getting wet they form a probiotic layer to outcompete the bad guys
  - Imaging of whole cities as opposed to spot sampling
  - Air filters with microbes – biofilter; Biological air cleaners to counteract viral infection
  - Build moisture problem free houses
  - Smart buildings that tell us when they are microbially compromised
- What needs to happen to get there: What are the good guys and what are the bad guys … and why are they … and what are they doing? Understand the genetic compound.

Other points that were noted as being relevant during this discussion were:

**Many houses with moisture problems that do not seem to be a health problem. What makes the difference?**

- Maybe there is a healthy indoor microbiome promoted by the moisture? Make observations on the exceptions. Is there something like healthy moldy buildings?
- Location is relevant. Air leakages.
- People evolve with their homes. And homes with their people.

**How clonal is fungal growth in a building?**

- Evolution of indoor fungal strains eg. to use biocides over decades?
- Apply selective pressure on fungi

**Longitudinal observations are of key importance!**

**Are antibiotics in buildings (produced by microbes) health relevant?**

**Some identified research needs:**

- Bacteria and fungal (co)occurrence and behaviour in indoor environments is understudied and poorly understood and they are usually quite different In some (many) cases, bacteria have found in undefined associations with fungi in the built environment, especially on moisture damaged materials.
- Moisture is the strongest environmental determinant of the indoor fungal and bacterial populations, and their interactions thereof
